# Supplementary material for: Accurate nowcasting of cloud cover at solar photovoltaic plants using geostationary satellite images
Source: Nat Commun. 2024 Jan 13;15:510. doi: 10.1038/s41467-023-44666-1 (PMC10787801; doi:10.1038/s41467-023-44666-1)
Supplement: Supplementary file 1 — Supplementary Information [file 41467_2023_44666_MOESM1_ESM.pdf]

Supplementary information for

Accurate nowcasting of cloud cover at solar photovoltaic plants using geostationary satellite images

Pan Xia[1], Lu Zhang[2], Min Min[1*], Jun Li[2], Yun Wang[3], Yu Yu[4], Shengjie Jia[5]

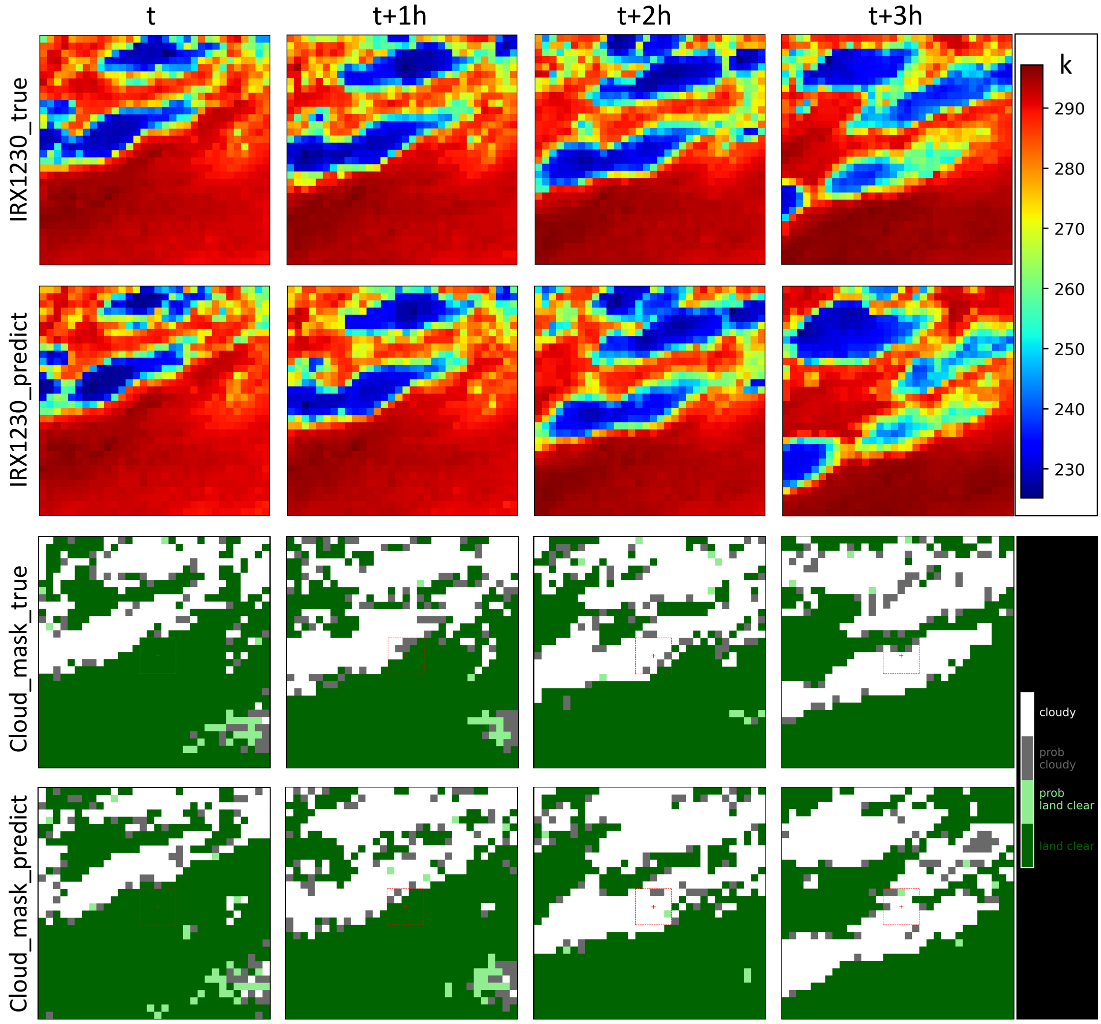

Supplementary Figure 1. Comparisons of true and predict satellite images (cloud mask products). Comparisons of observed (top row panel) and predicted satellite images at AHI 12.30 μm band (IRX1230, second row panels) (the colorbar represents brightness temperature (unit: Kelvin)) and the cloud mask products retrieved by actual observations (third row panel) and the predict cloud mask products retrieved by the predicted image data (bottom row panel) at Zhuhai station (red dashed square boxes) at UTC 05:00 (t, first column), 06:00 (t + 1 h, second column), 07:00 (t + 2 h, third column) and 08:00 (t + 3 h, fourth column) on November 17, 2022. The white, gray, light green and dark green spots represent cloudy, probably cloudy, probably clear and clear pixel labels, respectively.

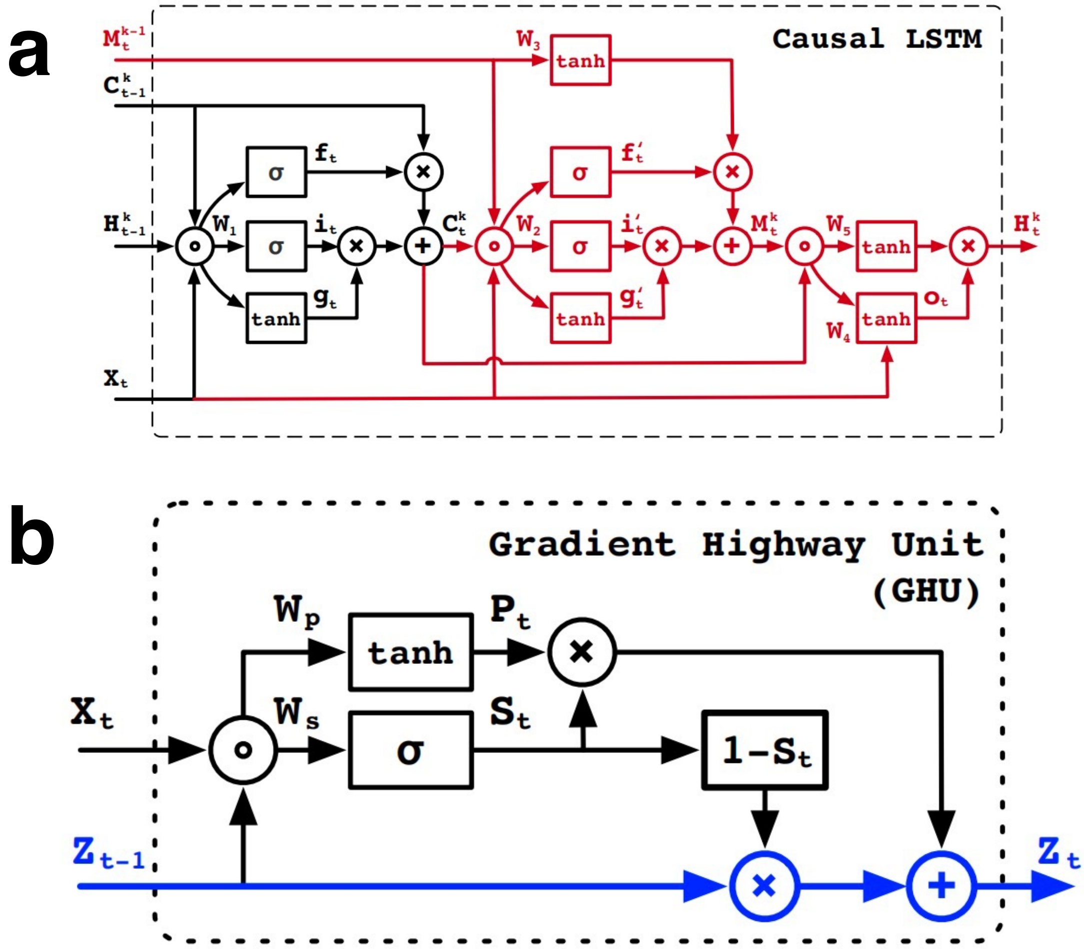

Supplementary Figure 2. (a) [1] **Causal long short-term memory (LSTM) and (b)** [1] **GHU.** The temporal (black) and spatial (red) memories are connected in a cascaded way through gated structures. "⊙" denotes the concatenation, "⊗" an element-wise multiplication, "⊕" an addition, and σ an element-wise sigmoid function. This figure has been adopted from Proceedings of Machine Learning Research (2018) with permission from the publisher. Reprinted with permission.

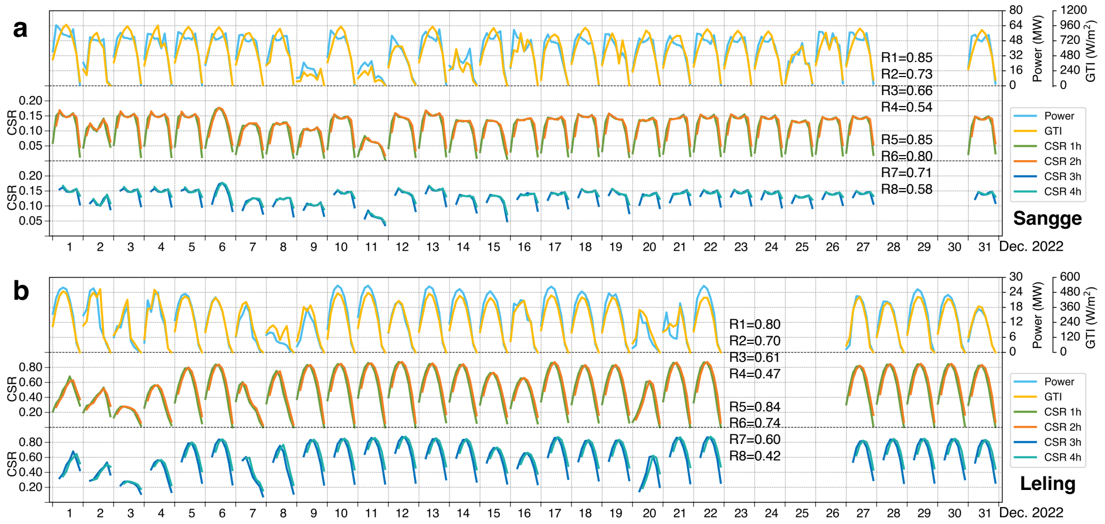

Supplementary Figure 3. The power and GTI of PV plants and corresponding 0 – 4 h CSR. Time series of the power (MW), GTI (W·m^{-2}) and predicted clear sky ratio (CSR) at Sangge (**a**), Leling (**b**) PV plants from 09:00 to 17:00 (local time or Beijing time) on each day in December 2022. R1 – R4 and R5 – R8 indicate the Rs of the predicted CSR with the power and the GTI for forecast leading time of 1 – 4 hours, respectively. Note that the missing and invalid data are not shown. The results for other months are shown in Figs. S3 – S6 of the supplementary materials. Source data are provided as a Source Data file.

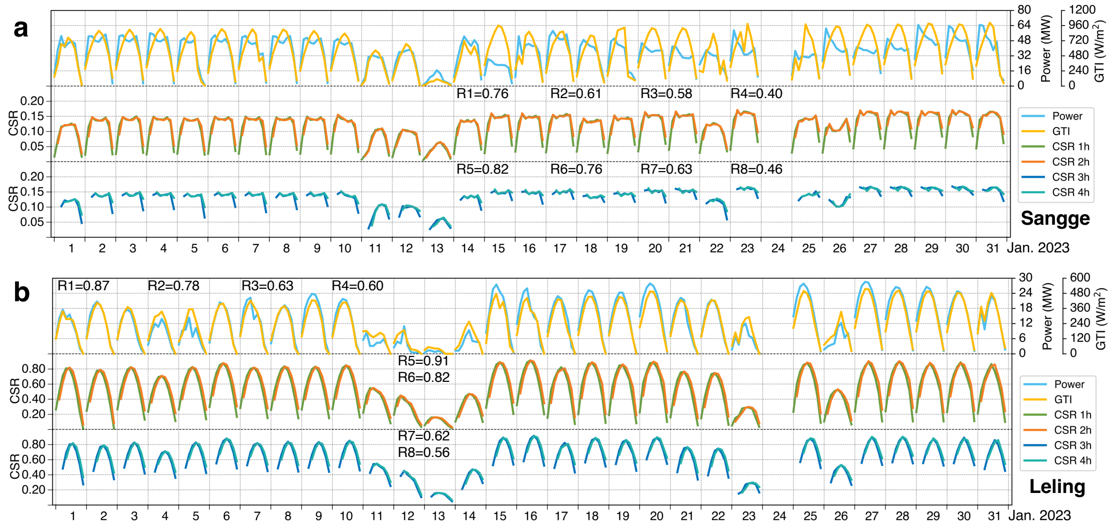

Supplementary Figure 4. Same as Supplementary Figure 3, but for the samples in January 2023. Source data are provided as a Source Data file.

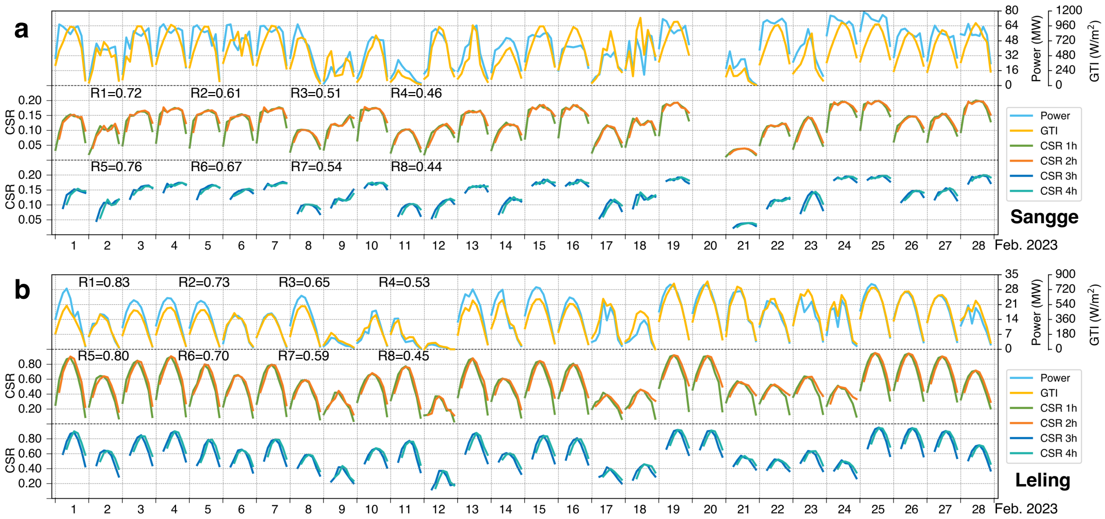

Supplementary Figure 5. Same as Supplementary Figure 3, but for the samples in February 2023. Source data are provided as a Source Data file.

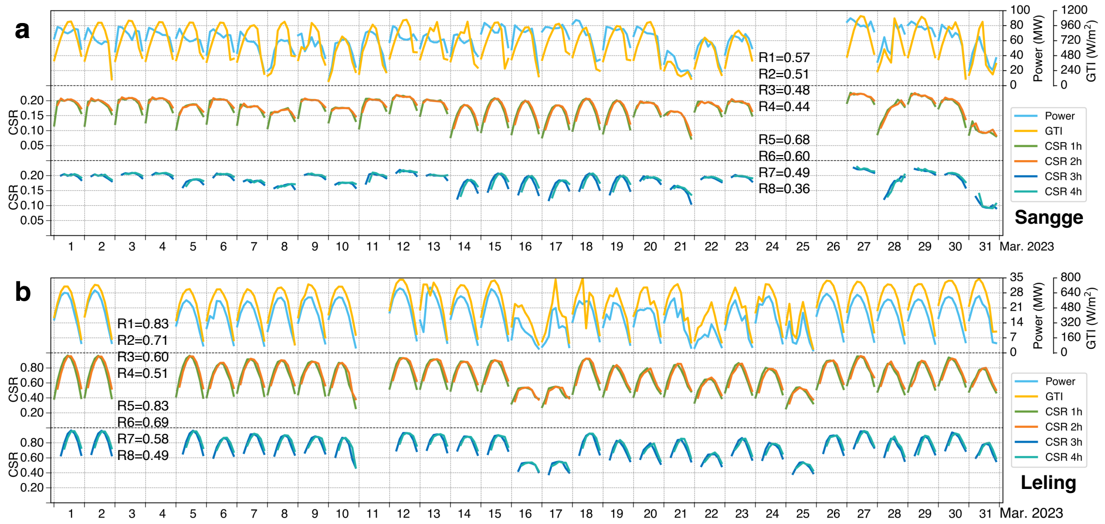

Supplementary Figure 6. Same as Supplementary Figure 3, but for the samples in March 2023. Source data are provided as a Source Data file.

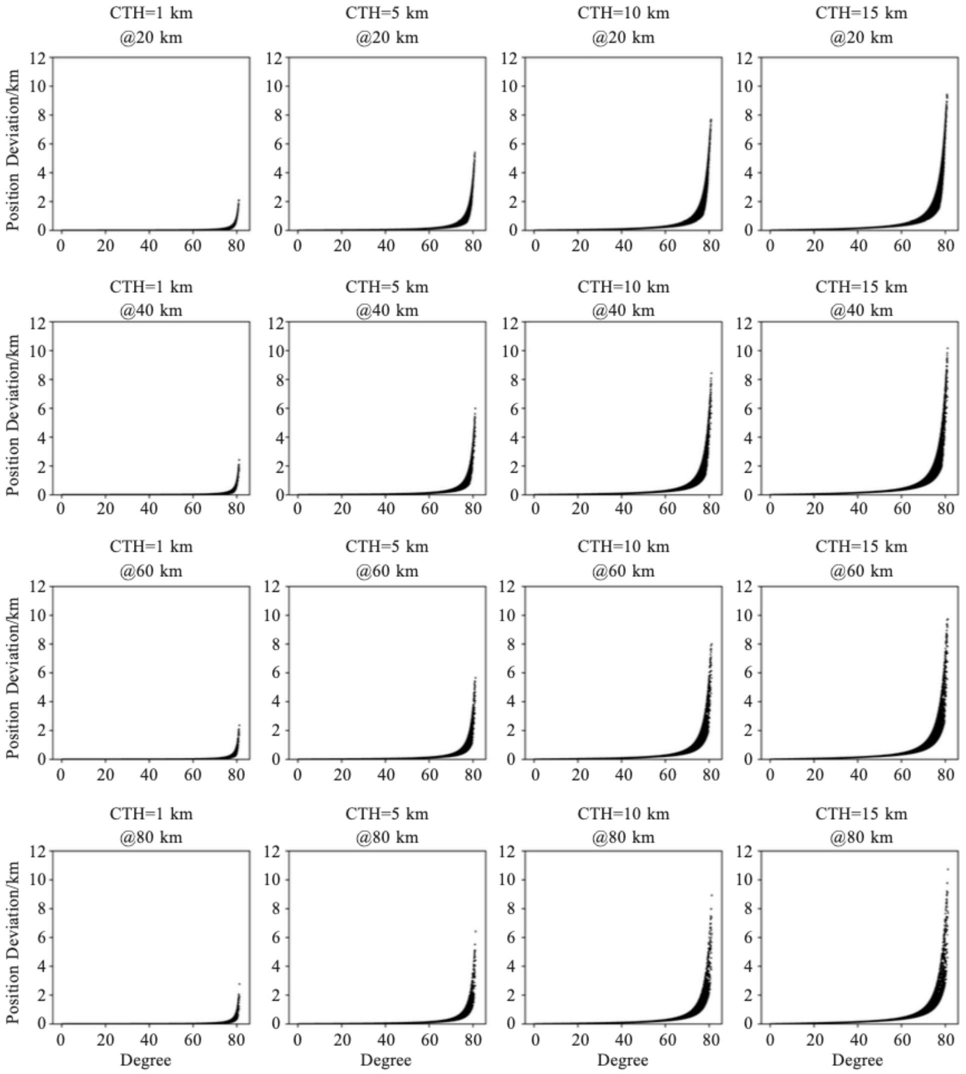

Supplementary Figure 7 [2]**. Sensitivity of position deviation (or parallax correction) to satellite imaging pixel spatial resolution, cloud top height (CTH), and view zenith angle using parallax correction algorithm.** This figure has been adopted from Journal of Tropical Meteorology (2022) with permission from the publisher. Reprinted with permission.

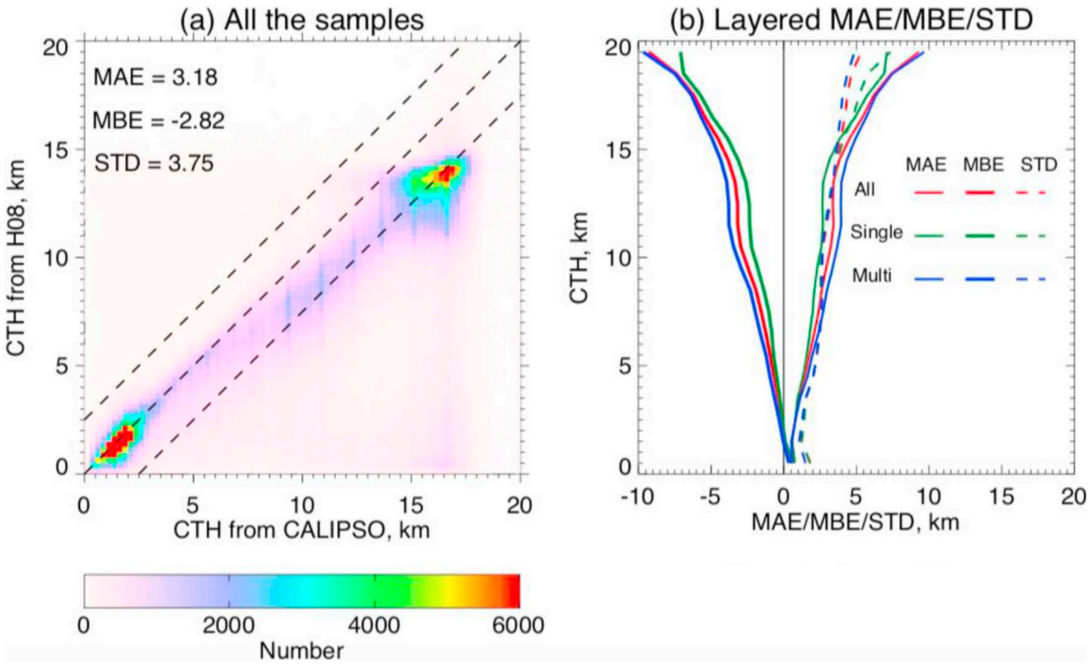

Supplementary Figure 8 [3]**. Validation (MAE=mean absolute error; MBE=mean bias error; STD=standard deviation) of cloud top height (CTH) retrieved from Himawari-8 satellite using CALIPSO (Cloud-Aerosol Lidar and Infrared Pathfinder Satellite Observation) product.** The color bar represents the total number in every bin at an interval of 0.25km.

Supplementary Note: We think the theoretical studies from Reference-1 (Wei and Sun 2022) mentioned above can help us to explain the relatively small or negligible parallax effect. Supplementary Figure 7 shows the sensitivity of position deviation (or parallax correction) to satellite imaging pixel spatial resolution, cloud top height (CTH), and view zenith angle using parallax correction algorithm (Wei and Sun 2022). First, it clearly proves that the position deviation is not sensitive to satellite imaging pixel spatial resolution. Second, the specific view zenith angles of ground-based stations used in this study from Supplementary Table 2 are not larger than 60 degrees (the maximum value is about 56 degrees). This figure also indicates that the bias caused by parallax is unlikely to exceed 1km under the condition of view zenith angle<60 degrees. Parallax correction should only be applied to exceptionally high clouds (>10km) resulting from

typhoons or Deep Convective Clouds with a view zenith angle greater than 70 degrees in the GEO satellite field of view.

In addition, it is worth noting that the parallax correction for GEO satellite images needs high-precision cloud top height products. Cloud top height products still manifest a notable degree of error, particularly in the case of relatively high cloud top samples with relatively larger retrieval errors (exceeding 4 km when cloud top height exceeds 10 km, as illustrated in Supplementary Figure 8). This may introduce more uncontrollable errors to the parallax correction algorithm.

Supplementary Table 1. MBE and RMSE between cloud fraction prediction results and manual observations at different forecast horizon. Source data are provided as a Source Data file.

Station	1h		2h		3h		4h	
	MBE	RMSE	MBE	RMSE	MBE	RMSE	MBE	RMSE
Hailisu	0.03256	0.22325	0.02895	0.26958	-0.0012	0.31066	0.03862	0.34259
Liupanshan	-0.04537	0.24666	-0.05423	0.28695	-0.05407	0.32912	-0.04431	0.35657
Xilinhot	0.08632	0.26918	0.04201	0.30063	0.0752	0.34329	0.07927	0.37779
Changchun	0.02969	0.2767	0.00056	0.31907	0.00854	0.35496	0.00285	0.38825
Miyun	-0.01819	0.23676	-0.00595	0.29076	-0.00809	0.32905	0.02504	0.36319
Chengtoushan	-0.02679	0.23282	-0.03936	0.28175	-0.04419	0.33722	-0.01274	0.36239
Songshan	-0.01179	0.20495	-0.04906	0.25389	-0.05057	0.29714	-0.05801	0.34374
Xiaogan	0.09024	0.22986	0.07312	0.27931	0.06077	0.305	0.08475	0.32827
Youyang	-0.01127	0.16686	-0.04715	0.21286	-0.06077	0.24932	-0.06985	0.29385
Hangzhou	0.08225	0.22801	0.07797	0.28728	0.07855	0.31434	0.0737	0.3391
Chongwu	0.04786	0.24414	0.06828	0.30888	0.10071	0.35635	0.10416	0.38932
Zengcheng	0.06663	0.24232	0.03615	0.26559	0.04877	0.29168	0.06033	0.3244

Supplementary Table 2. Satellite view zenith angle of PV plants and All-sky imager stations.

Station	Satellite view zenith angle (H8/9)	Longitude, Lattitude
Sangge	56.38°	109.71° E, 40.30° N
Leling	50.05°	117.28° E, 37.67° N
Xiaochengzi	52.47°	119.31° E, 41.29° N
Lijiamen	41.36°	111.99° E, 29.72° N
Shiziyan	44.81°	114.68° E, 29.68° N
Beijing	52.48°	116.43° E, 39.91° N
Nanjing	44.15°	118.96° E, 32.11° N
Zhuhai	40.25°	113.58° E, 22.35° N

Supplementary References

1. Wang Y, Gao Z, Long M, Wang J, Yu PS. PredRNN++: Towards a resolution of the deep-in-time dilemma in spatiotemporal predictive learning. In: *Proceedings of the 35th International Conference on Machine Learning*). PMLR (2018).
2. Xiaocheng Wei, Fenglin Sun. Analysis of the parallax characteristics of geostationary-orbiting microwave sounder [J]. *Journal of Tropical Meteorology*, 38(6): 901-914, (2022). doi: 10.16032/j.issn.1004-4965.2022.067.
3. Min Min, Jun Li, Fu Wang, Zijing Liu, W. Paul Menzel. Retrieval of cloud top properties from advanced geostationary satellite imager measurements based on machine learning algorithms [J]. *Remote Sensing of Environment*, 239: 111616, (2020). doi: 10.1016/j.rse.2019.111616.
