# Supplementary material for: Accurate nowcasting of cloud cover at solar photovoltaic plants using geostationary satellite images
Source: Nat Commun. 2024 Jan 13;15:510. doi: 10.1038/s41467-023-44666-1 (PMC10787801; doi:10.1038/s41467-023-44666-1)
Supplement: Supplementary file 3 — Reporting Summary [file 41467_2023_44666_MOESM3_ESM.pdf]

nature portfolio

Corresponding author(s): Min Min

Last updated by author(s): Oct 4, 2023

Reporting Summary

Nature Portfolio wishes to improve the reproducibility of the work that we publish. This form provides structure for consistency and transparency in reporting. For further information on Nature Portfolio policies, see our Editorial Policies and the Editorial Policy Checklist.

Statistics

For all statistical analyses, confirm that the following items are present in the figure legend, table legend, main text, or Methods section.

n/a	Confirmed	
	x	The exact sample size (*n*) for each experimental group/condition, given as a discrete number and unit of measurement
	x	A statement on whether measurements were taken from distinct samples or whether the same sample was measured repeatedly
x		The statistical test(s) used AND whether they are one- or two-sided<br>*Only common tests should be described solely by name; describe more complex techniques in the Methods section.*
x		A description of all covariates tested
x		A description of any assumptions or corrections, such as tests of normality and adjustment for multiple comparisons
x		A full description of the statistical parameters including central tendency (e.g. means) or other basic estimates (e.g. regression coefficient) AND variation (e.g. standard deviation) or associated estimates of uncertainty (e.g. confidence intervals)
x		For null hypothesis testing, the test statistic (e.g. *F*, *t*, *r*) with confidence intervals, effect sizes, degrees of freedom and *P* value noted<br>*Give P values as exact values whenever suitable.*
x		For Bayesian analysis, information on the choice of priors and Markov chain Monte Carlo settings
x		For hierarchical and complex designs, identification of the appropriate level for tests and full reporting of outcomes
	x	Estimates of effect sizes (e.g. Cohen's *d*, Pearson's *r*), indicating how they were calculated

Our web collection on statistics for biologists contains articles on many of the points above.

Software and code

Policy information about availability of computer code

Data collection	The Himawari-8/9 data are available for free download from website [http://www.jma-net.go.jp/msc/en/]. Source data are provided with this paper.
Data analysis	We appreciate the Facebook Inc. for freely providing the Pytorch toolkit (https://pytorch.org).

For manuscripts utilizing custom algorithms or software that are central to the research but not yet described in published literature, software must be made available to editors and reviewers. We strongly encourage code deposition in a community repository (e.g. GitHub). See the Nature Portfolio guidelines for submitting code & software for further information.

Data

Policy information about availability of data

All manuscripts must include a data availability statement. This statement should provide the following information, where applicable:

- Accession codes, unique identifiers, or web links for publicly available datasets
- A description of any restrictions on data availability
- For clinical datasets or third party data, please ensure that the statement adheres to our policy

The Himawari-8/9 data are available for free download from website [http://www.jma-net.go.jp/msc/en/]. Source data are provided with this paper.

Research involving human participants, their data, or biological material

Policy information about studies with human participants or human data. See also policy information about sex, gender (identity/presentation), and sexual orientation and race, ethnicity and racism.

Reporting on sex and gender	n/a
Reporting on race, ethnicity, or other socially relevant groupings	n/a
Population characteristics	n/a
Recruitment	n/a
Ethics oversight	n/a

Note that full information on the approval of the study protocol must also be provided in the manuscript.

Field-specific reporting

Please select the one below that is the best fit for your research. If you are not sure, read the appropriate sections before making your selection.

☐ Life sciences ☐ Behavioural & social sciences ☒ Ecological, evolutionary & environmental sciences

For a reference copy of the document with all sections, see nature.com/documents/nr-reporting-summary-flat.pdf

Ecological, evolutionary & environmental sciences study design

All studies must disclose on these points even when the disclosure is negative.

Study description	Accurate nowcasting for cloud fraction (CF) is still intractable challenge for stable solar photovoltaic (PV) electricity generation. By combining continuous radiance images measured by geostationary satellite and an advanced recurrent neural network, we develop a nowcasting algorithm for predicting CF at the leading time of 0–4 hours at PV plants. Based on this algorithm, a cyclically updated prediction system is also established and tested at five PV plants and several stations with CF observations in China. The results demonstrate that the CF nowcasting is efficient, high quality and adaptable. Particularly, it shows an excellent forecast performance within the first 2-hour leading time, with an average correlation coefficient close to 0.8 between the predicted clear sky ratio (CSR) and actual power generation at PV plants. Our findings highlight the benefits and potential of this technique to improve the competitiveness of solar PV energy in electricity market.
Research sample	A cyclically updated prediction system is also established and tested at five PV plants and several stations with CF observations in China.
Sampling strategy	n/a
Data collection	1. Geostationary satellite data and calculating the cloud fraction. The NRT 16-band full-disk AHI level-1B radiance data from the Himawari-8/9 satellite (the new-generation Japanese geostationary meteorological satellite) with spatio-temporal resolutions of 1 – 4 km and 10 minutes are obtained from the Japan Meteorological Agency Himawari-Cast in China 28. Additionally, the offline Himawari-8/9 data at the original resolution (0.5 – 2 km) are also available for free download from the JAXA (Japan Aerospace Exploration Agency) Himawari satellite data FTP (File Transfer Protocol) site (ftp.ptree.jaxa.jp) from July 7, 2015 (http://www.jma-net.go.jp/msc/en/). The nadir point of the Himawari-8/9 satellite is located at 140.7°E, and the coverage of this satellite includes the Japan island and the eastern parts of China.<br><br>2. Ground-based observation data. The total cloud cover or CF (about 20 km × 20 km square area) used in this study is obtained through manual observation at 12 ground-based meteorological stations (Fig. 1) in January, April, July and October of 2019. Note that due to the relatively large errors in low-visibility conditions, the CF data with the matched and automatically measured visibility less than 2 km is removed. Besides, the view zenith angles of ground-based stations from H8/AHI field of view used here, as stated in Supplementary Table 2, are smaller than 60°. Therefore, the parallax effect is negligible (error<1km) in the collocation between satellite pixels and ground-based stations for this study. Firstly, considering the daytime nowcasting applications and sunshine conditions at PV plants, only the manual observations at 11:00, 14:00 and 17:00 are collected for validation in this research. Secondly, three ground-based all-sky imager stations (equipped with a Japan EKO ASI-16 all-sky imager, https://www.eko-instruments.com/us/categories/products/all-sky-imagers/asi-16-all-sky-imager) can provide the high temporal resolution (5 minutes) and valuable CF and cloud cover data during the daytime, which are retrieved by the standard EKO ASI-16 cloud detection algorithm (https://www.eko-instruments.com/media/z2aalysq/asi-16-software-manual-find-clouds.pdf) 32. ASI, equipped with a digital camera coupled with an upward looking fisheye lens, could provide field of view (FOV) of about 180º, but pixels at a FOV >140 º are excluded due to distortion. Digital images of the sky obtained by ASI are classified pixel by pixel into clear sky, optically thin and optically thick clouds, respectively. The cloud detection and opacity classification (CDOC) algorithm developed by Ghonima et al., 2012 32 could provide 96%, 60%, and 96.3% accuracy in the validation for clear, thin, and thick cloud, respectively. Finer CF data allow more accurate validation of the NCP_CF system, thereby demonstrating the specific prediction effect at the forecast leading time of 0–4 hours. The study periods of the local CF data from three all-sky imagers located in Zhuhai, Nanjing and Beijing (Fig. 1) are from September 2022 to February 2023, April 2021 to September 2021, and September 2022 to December 2022, respectively. In addition,

	the actual power generation (MW, temporal resolution of 15 minutes) and GTI (W·m−2) measured at Sangge and Leling PV plants from November 2022 to March 2023 and at Xiaochengzi, Lijiamen and Shiziyan PV plants in November 2022 (Fig. 1) are also used to analyze the agreement with the predicted CF.
Timing and spatial scale	n/a
Data exclusions	n/a
Reproducibility	n/a
Randomization	n/a
Blinding	n/a

Did the study involve field work? ☒ Yes ☐ No

Field work, collection and transport

Field conditions	cloud fraction
Location	China
Access & import/export	n/a
Disturbance	n/a

Reporting for specific materials, systems and methods

We require information from authors about some types of materials, experimental systems and methods used in many studies. Here, indicate whether each material, system or method listed is relevant to your study. If you are not sure if a list item applies to your research, read the appropriate section before selecting a response.

Materials & experimental systems

n/a	Involved in the study	
☒	☐	Antibodies
☒	☐	Eukaryotic cell lines
☒	☐	Palaeontology and archaeology
☒	☐	Animals and other organisms
☒	☐	Clinical data
☒	☐	Dual use research of concern
☒	☐	Plants

Methods

n/a	Involved in the study	
☒	☐	ChIP-seq
☒	☐	Flow cytometry
☒	☐	MRI-based neuroimaging
