# Supplementary material for: Accurate nowcasting of cloud cover at solar photovoltaic plants using geostationary satellite images
Source: Nat Commun. 2024 Jan 13;15:510. doi: 10.1038/s41467-023-44666-1 (PMC10787801; doi:10.1038/s41467-023-44666-1)
Supplement: Supplementary file 4 — Source data [file 41467_2023_44666_MOESM4_ESM.zip › Source_Data/Source_Data_Figure1/Latitude and longitude of all sites.docx]

**Manual observation station**

| name | Latitude (N) | Longitude (E) |
| --- | --- | --- |
| Hailisu | 41.4 | 106.4 |
| Liupanshan | 35.667 | 106.2 |
| Xilinhot | 43.95 | 116.117 |
| Changchun | 43.9 | 125.217 |
| Miyun | 40.308 | 116.867 |
| Chengtoushan | 37.4 | 122.7 |
| Songshan | 34.5 | 113.05 |
| Xiaogan | 30.9 | 113.95 |
| Youyang | 28.817 | 108.767 |
| Hangzhou | 30.233 | 120.167 |
| Chongwu | 24.9 | 118.917 |
| Zengcheng | 23.333 | 113.833 |

**All-sky imager station**

| name | Latitude (N) | Longitude (E) |
| --- | --- | --- |
| Beijing | 39.9055 | 116.425 |
| Nanjing | 32.1086 | 118.9596 |
| Zhuhai | 22.35 | 113.58 |

**PV power test plant**

| name | Latitude (N) | Longitude (E) |
| --- | --- | --- |
| Sangge | 40.299 | 109.71 |
| Leling | 37.6667 | 117.2833 |
| Xiaochengzi | 41.2971 | 119.3182 |
| Lijiamen | 29.7183 | 111.9954 |
| Shiziyan | 29.6784 | 114.6849 |
