# Supplementary figures and images for: Accurate nowcasting of cloud cover at solar photovoltaic plants using geostationary satellite images
Source: Nat Commun. 2024 Jan 13;15:510. doi: 10.1038/s41467-023-44666-1 (PMC10787801; doi:10.1038/s41467-023-44666-1)

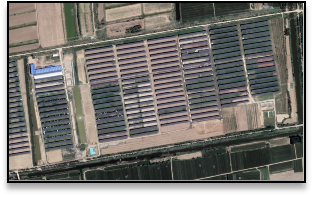

Supplement: Supplementary file 4 — Source data [file 41467_2023_44666_MOESM4_ESM.zip › Source_Data/Source_Data_Figure1/Leling.png]

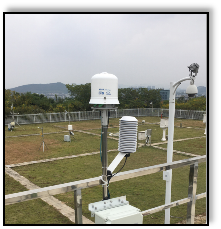

Supplement: Supplementary file 4 — Source data [file 41467_2023_44666_MOESM4_ESM.zip › Source_Data/Source_Data_Figure1/Nanjing.png]

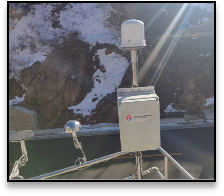

Supplement: Supplementary file 4 — Source data [file 41467_2023_44666_MOESM4_ESM.zip › Source_Data/Source_Data_Figure1/Beijing.png]

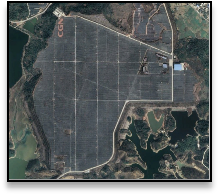

Supplement: Supplementary file 4 — Source data [file 41467_2023_44666_MOESM4_ESM.zip › Source_Data/Source_Data_Figure1/Shiziyan.png]

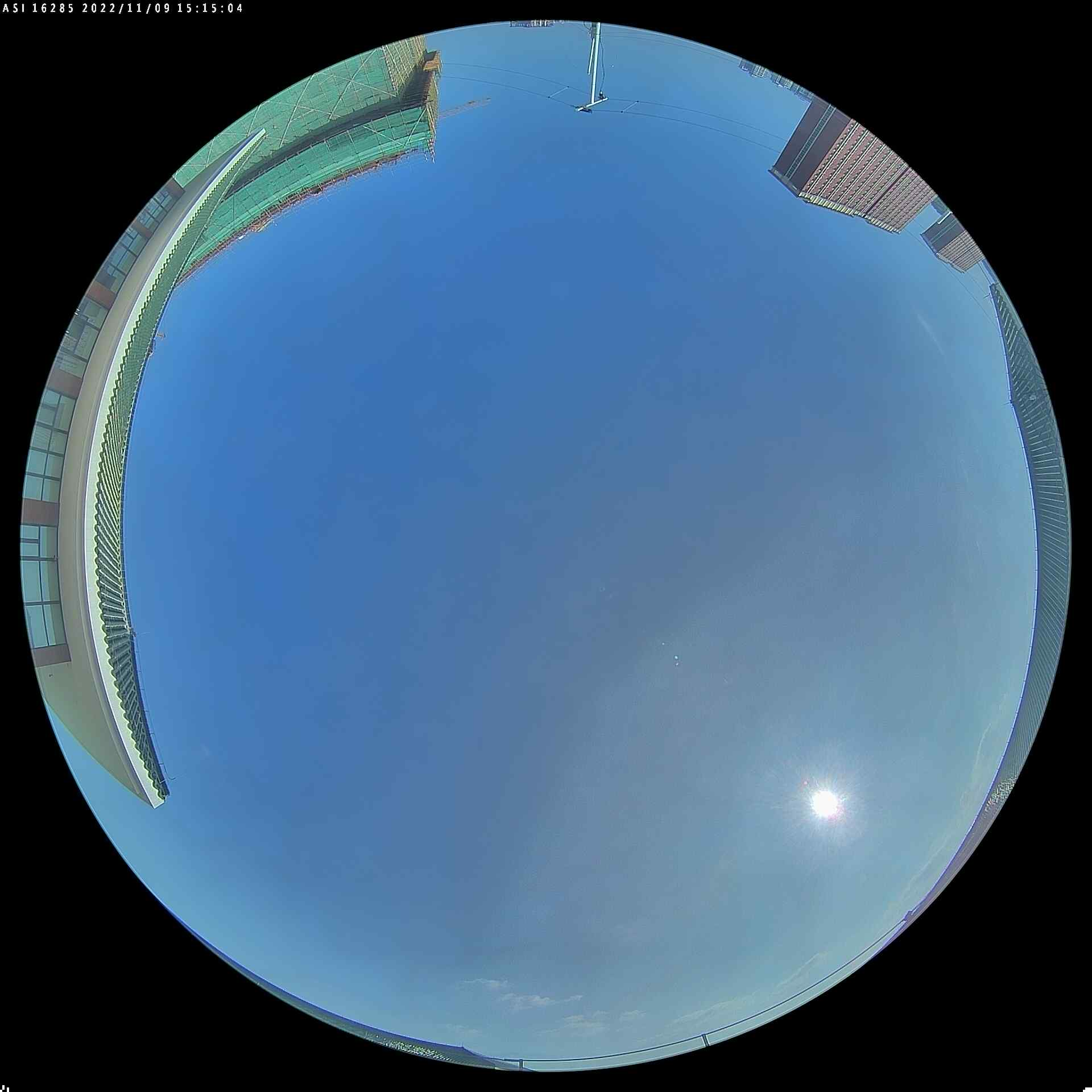

Supplement: Supplementary file 4 — Source data [file 41467_2023_44666_MOESM4_ESM.zip › Source_Data/Source_Data_Figure1/Zhuhai_all-sky_observation.png]

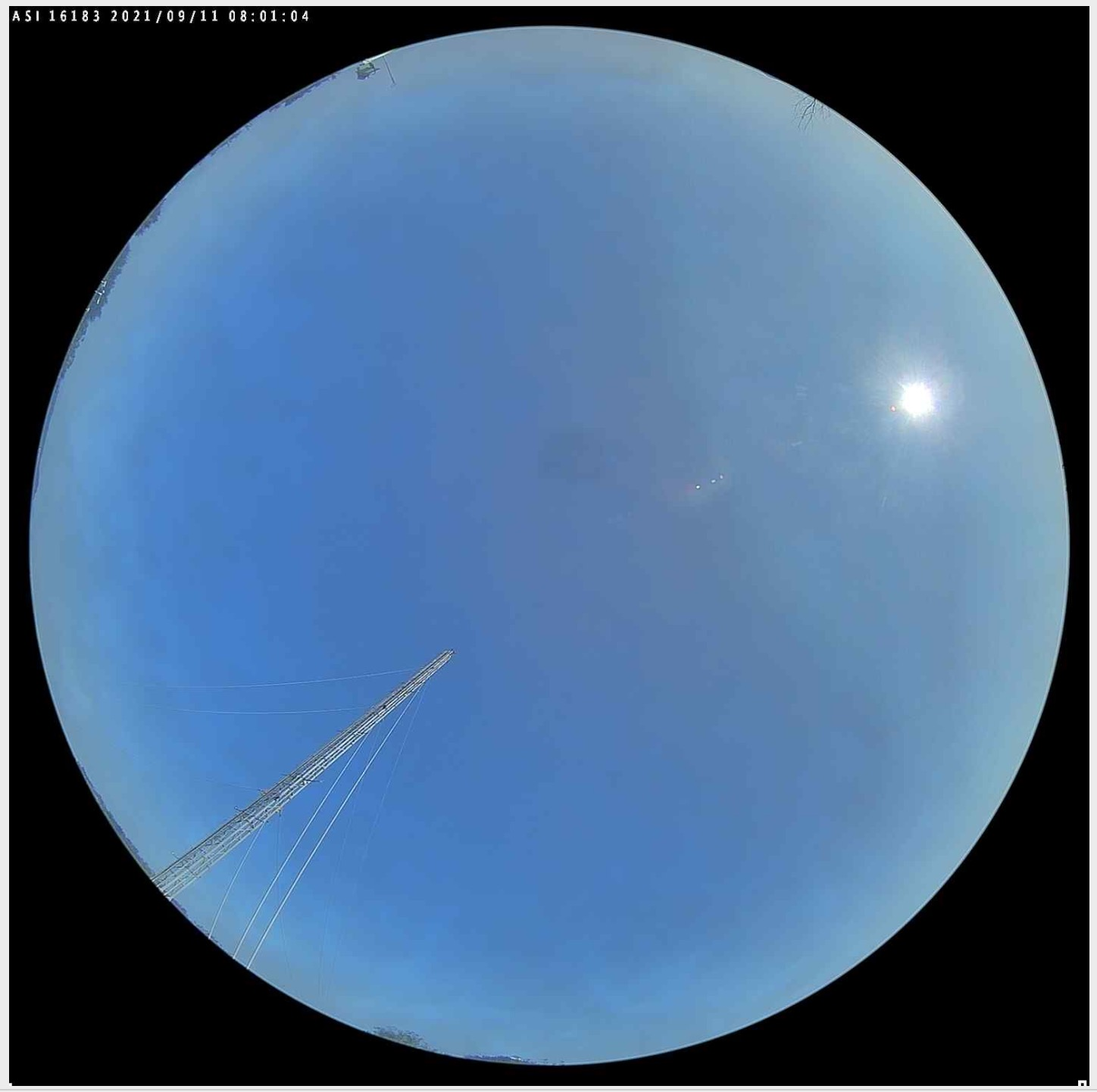

Supplement: Supplementary file 4 — Source data [file 41467_2023_44666_MOESM4_ESM.zip › Source_Data/Source_Data_Figure1/Nanjing_all-sky_observation.png]

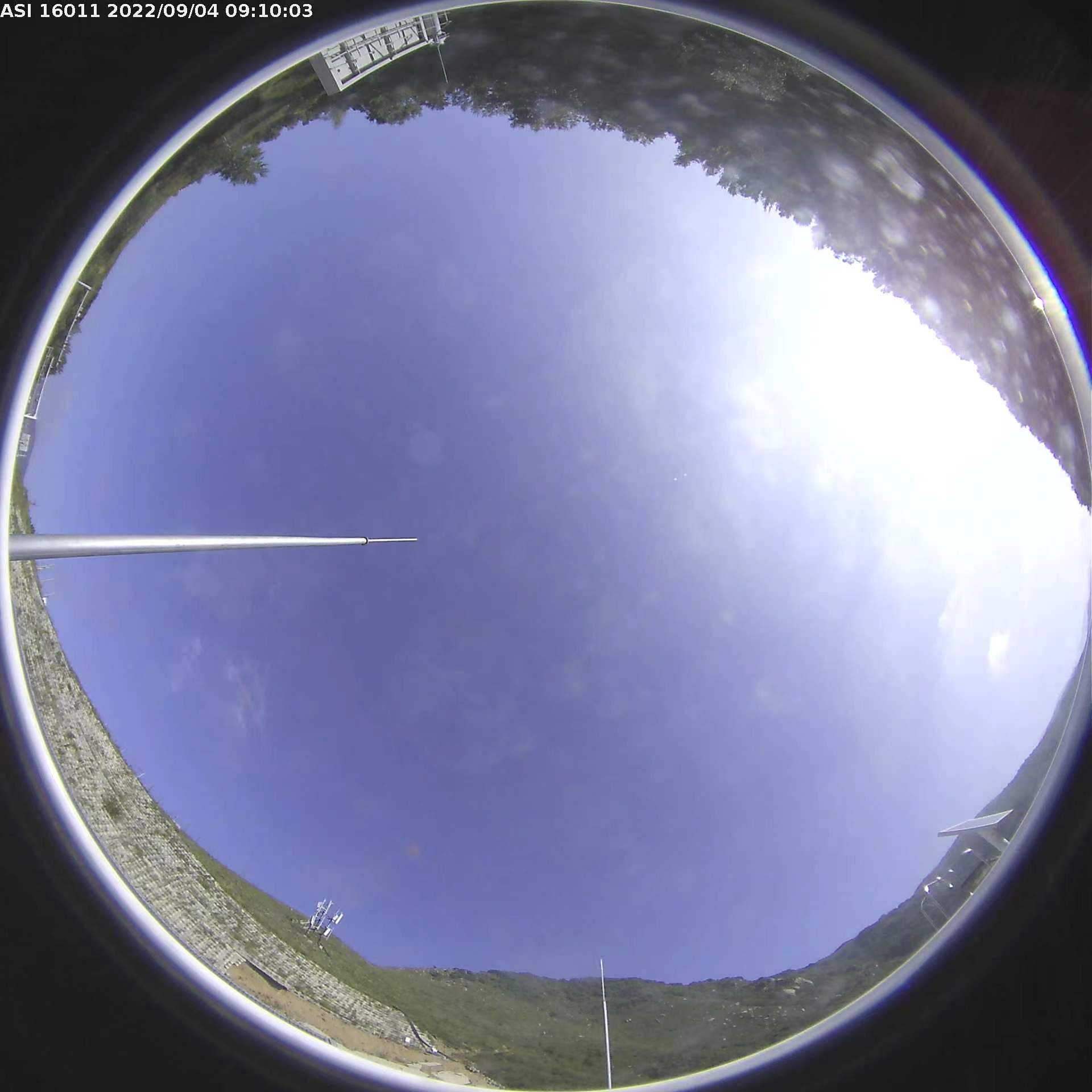

Supplement: Supplementary file 4 — Source data [file 41467_2023_44666_MOESM4_ESM.zip › Source_Data/Source_Data_Figure1/Beijing_all-sky_observation.png]

## Slide 1
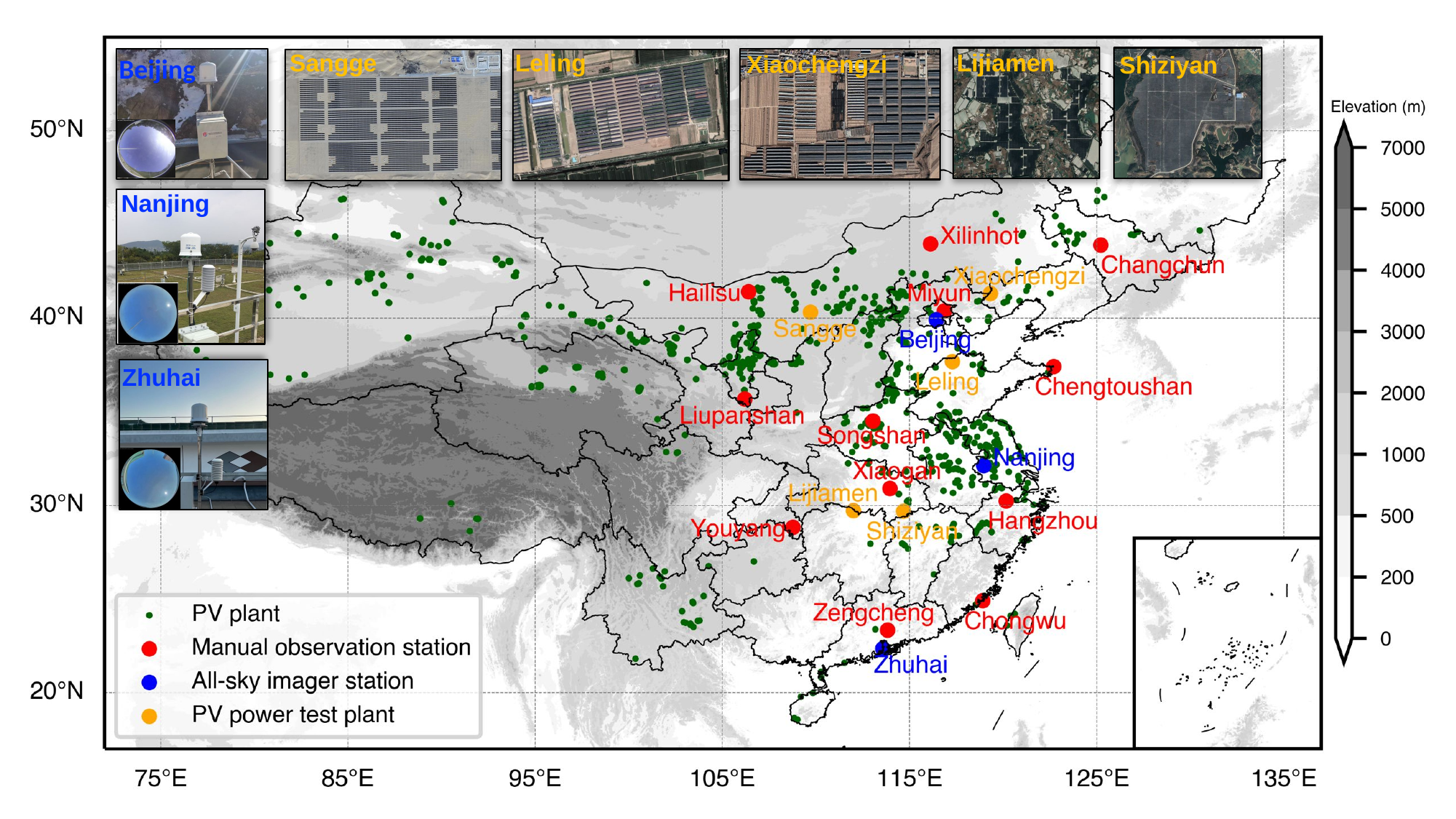

Sangge
Leling
Lijiamen
Xiaochengzi
Shiziyan
Beijing
Nanjing
Zhuhai

Supplement: Supplementary file 4 — Source data [file 41467_2023_44666_MOESM4_ESM.zip › Source_Data/Source_Data_Figure1/Figure1_r1_new.pptx]

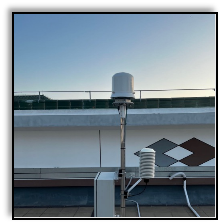

Supplement: Supplementary file 4 — Source data [file 41467_2023_44666_MOESM4_ESM.zip › Source_Data/Source_Data_Figure1/Zhuhai.png]

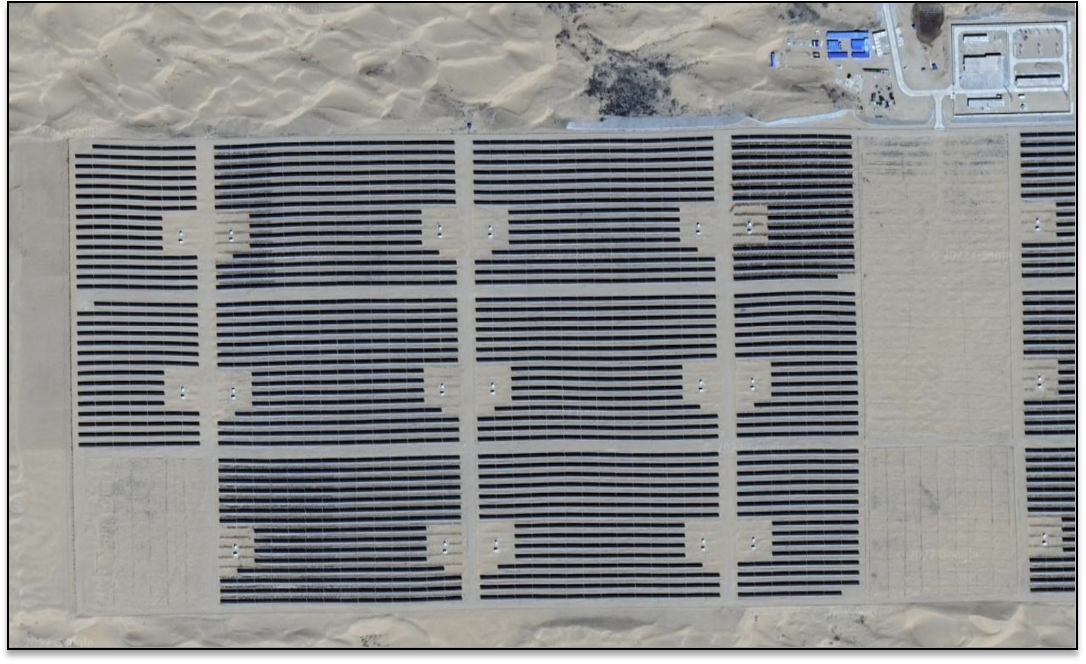

Supplement: Supplementary file 4 — Source data [file 41467_2023_44666_MOESM4_ESM.zip › Source_Data/Source_Data_Figure1/Sangge.png]

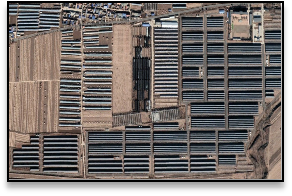

Supplement: Supplementary file 4 — Source data [file 41467_2023_44666_MOESM4_ESM.zip › Source_Data/Source_Data_Figure1/Xiaochengzi.png]

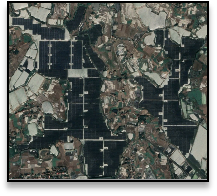

Supplement: Supplementary file 4 — Source data [file 41467_2023_44666_MOESM4_ESM.zip › Source_Data/Source_Data_Figure1/Lijiamen.png]
